# Supplementary material for: Exposure to Gold Induces Autoantibodies against Nuclear Antigens in A.TL Mice
Source: Biology (Basel). 2024 Oct 11;13(10):812. doi: 10.3390/biology13100812 (PMC11505499; doi:10.3390/biology13100812)
Supplement: Supplementary file 1 [file biology-13-00812-s001.zip › biology-3154941-supplementary.pdf]

Supplementary Figure S1

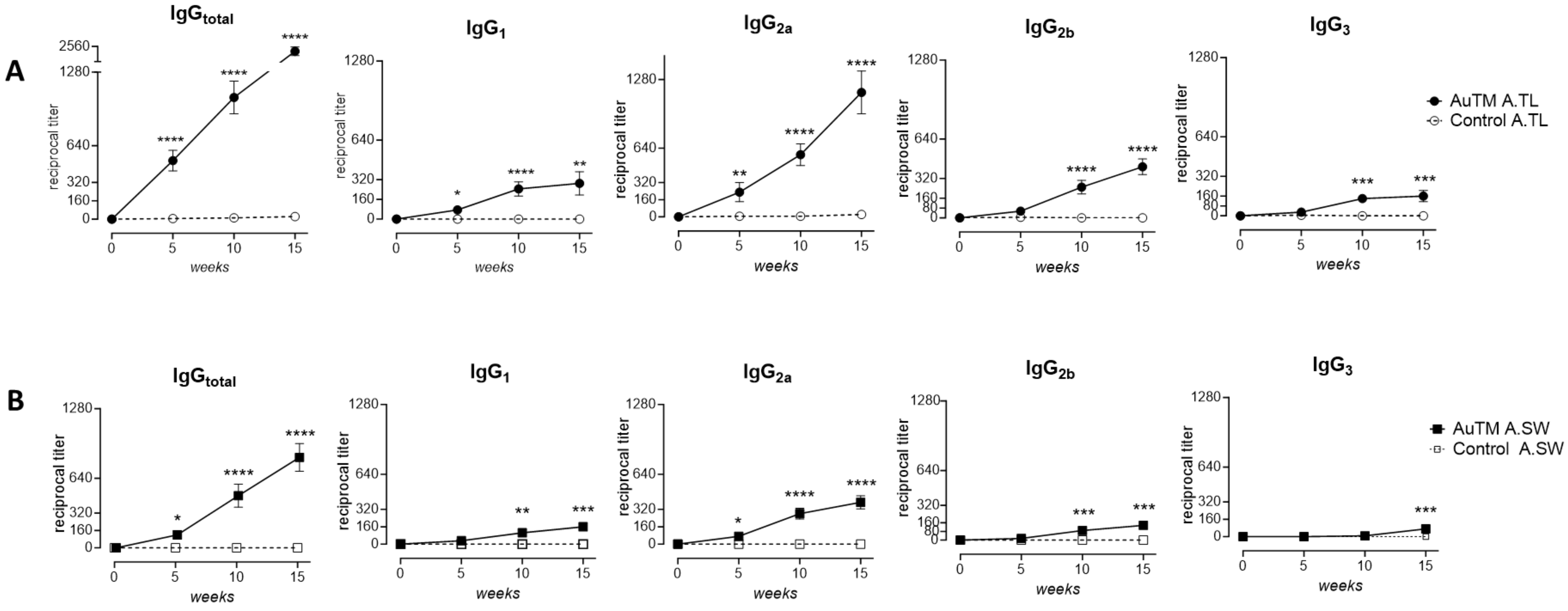

Figure S1. Titer of serum anti-nuclear antibodies (ANA) of AuTM and control mice. IgG total and IgG subclasses titers in **(A)** A.TL and **(B)** A.SW mice treated with AuTM or H_2O (control) detected by indirect immunofluorescence. Serum was incubated on HEp-2 cells and then detected with FITC-conjugated anti-mouse IgG antibodies. * = $p < 0.05$; ** = $p < 0.01$; ***= $p < 0.001$; ****=$p < 0.0001$ indicate differences between AuTM- and control-treated mice using Mann-Whitney U test. Error bars indicate SEM. n=number of mice, AuTM A.TL (n=16), Control A.TL (n=14), AuTM A.SW (n=10), Control A.SW (n=9).
